# Deep Learning Method for Breakdown Voltage and Forward *I-V* Characteristic Prediction of Silicon Carbide Schottky Barrier Diodes

**DOI:** 10.3390/mi16050583

**Published:** 2025-05-15

**Authors:** Hao Zhou, Xiang Wang, Shulong Wang, Chenyu Liu, Dongliang Chen, Jiarui Li, Lan Ma, Guohao Zhang

**Affiliations:** 1 School of Microelectronics, Xidian University, Xi’an 710071, China; hzhou.1@stu.xidian.edu.cn (H.Z.); lauchenyu@163.com (C.L.); dl.cymbidium@gmail.com (D.C.); 18234192977@163.com (J.L.); lanmaxd@163.com (L.M.); 2 School of Information Engineering, Guangdong University of Technology, Guangzhou 510006, China; wangxiang@sgchip.sgcc.com.cn (X.W.); garyzhang@gdut.edu.cn (G.Z.)

**Keywords:** SiC, breakdown voltage, deep learning, neural network

## Abstract

This work employs a deep learning method to develop a high-precision model for predicting the breakdown voltage (*V*_br_) and forward *I-V* characteristics of silicon carbide Schottky barrier diodes (SiC SBDs). The model significantly reduces the testing costs associated with destructive experiments, such as breakdown voltage testing. Although the model requires a certain amount of time to establish itself, it supports linear variations in related variables once developed. A predicted model for *V*_br_ with an accuracy of up to 99% was successfully developed using 600 sets of input data after 200 epochs of training. After training for 1000 epochs, the deep learning-based model could predict not only point values like *V*_br_ but also curves, such as forward *I-V* characteristics, with a mean squared error (MSE) of less than 10^−3^. Our research shows the applicability and high efficiency of introducing deep learning into device characteristic prediction.

## 1. Introduction

Silicon carbide (SiC) is increasingly favored for high-voltage applications because of its wide bandgap, high thermal conductivity, and strong radiation resistance [1,2,3]. The Schottky barrier diode (SBD) is a prominent power electronic device in high-voltage applications with mature production processes, and breakdown voltage (*V*_br_) is a critical parameter for SiC SBDs [4,5,6]. The wide bandgap (3.26 eV for 4H-SiC) enables critical electric fields 10× higher than silicon, determining the theoretical limit of *V*_br_ in SiC SBD [7]. However, the development of high-voltage SiC SBDs faces a critical bottleneck: each design iteration requires multiple destructive *V*_br_ tests, leading to the exponential growth of costs in the design-of-experiments (DOEs) phase. Researchers must prioritize the refinement of device modeling processes, wherein the application of technology computer-aided design (TCAD) techniques plays a pivotal role.

While TCAD simulations have partially addressed electric field optimization challenges, their inherent limitation lies in the single-output-per-simulation paradigm, which restricts the exploration of continuous parameter variations and their corresponding device performance trends. Moreover, the currently widely used semiconductor device models can be divided into compact models and physics models. Unlike physics models constructed based on semiconductor theories, compact models focus on the external behavior of devices, enabling a fast modeling process. However, due to the reliance on empirical formulae, compact model methods demonstrate limited capability in predicting device characteristics involving complex physical mechanisms. Meanwhile, although physical model methods can accurately simulate the physical mechanisms in devices through physical equations, this process is often time-consuming [8,9]. There is an urgent need for a high-precision model to be capable of accounting for more complex physical mechanisms while achieving accurate predictions of device performance.

Machine learning has demonstrated significant technological merits in the performance optimization of SiC power devices. However, current research predominantly focuses on the predictive modeling of univariate static parameters such as on-resistance and breakdown voltage, while intelligent modeling methodologies for *I-V* characteristic curves remain systematically unexplored. Notably, existing predictive frameworks predominantly employ conventional machine learning algorithms that demonstrate insufficient fitting accuracy and limited generalization capabilities when addressing transient characteristic curves with strong nonlinear features [10,11,12,13]. Recent advances in deep learning (DL), a subset of machine learning characterized by hierarchical feature abstraction, particularly convolutional neural networks (CNNs) [14,15,16,17], offer transformative potential for device modeling. By leveraging a special linear mathematical operation [18,19,20,21], CNN has shown remarkable advantages in image classification, object detection, and image segmentation tasks. Owing to its inherent advantages mentioned above, CNN architecture has significant potential for establishing high-accuracy performance prediction models of power semiconductor devices.

In this work, we propose a deep-learning-based approach to predict *V*_br_ in SiC SBDs. The simulation results of the *V*_br_ for SiC SBDs with junction terminal extension (JTE) and field-limiting ring (FLR) are used as the input for the prediction model. The trained prediction model supports the linear variation in the device’s structural parameters. In addition, our prediction model is capable of not only predicting breakdown voltage but also forward *I-V* characteristics with high precision. The proposed method enables a rapid performance assessment during device optimization and can simultaneously predict multiple key electrical characteristics.

## 2. Methodology

### 2.1. Device Structure and Data Collection

**Figure 1:**
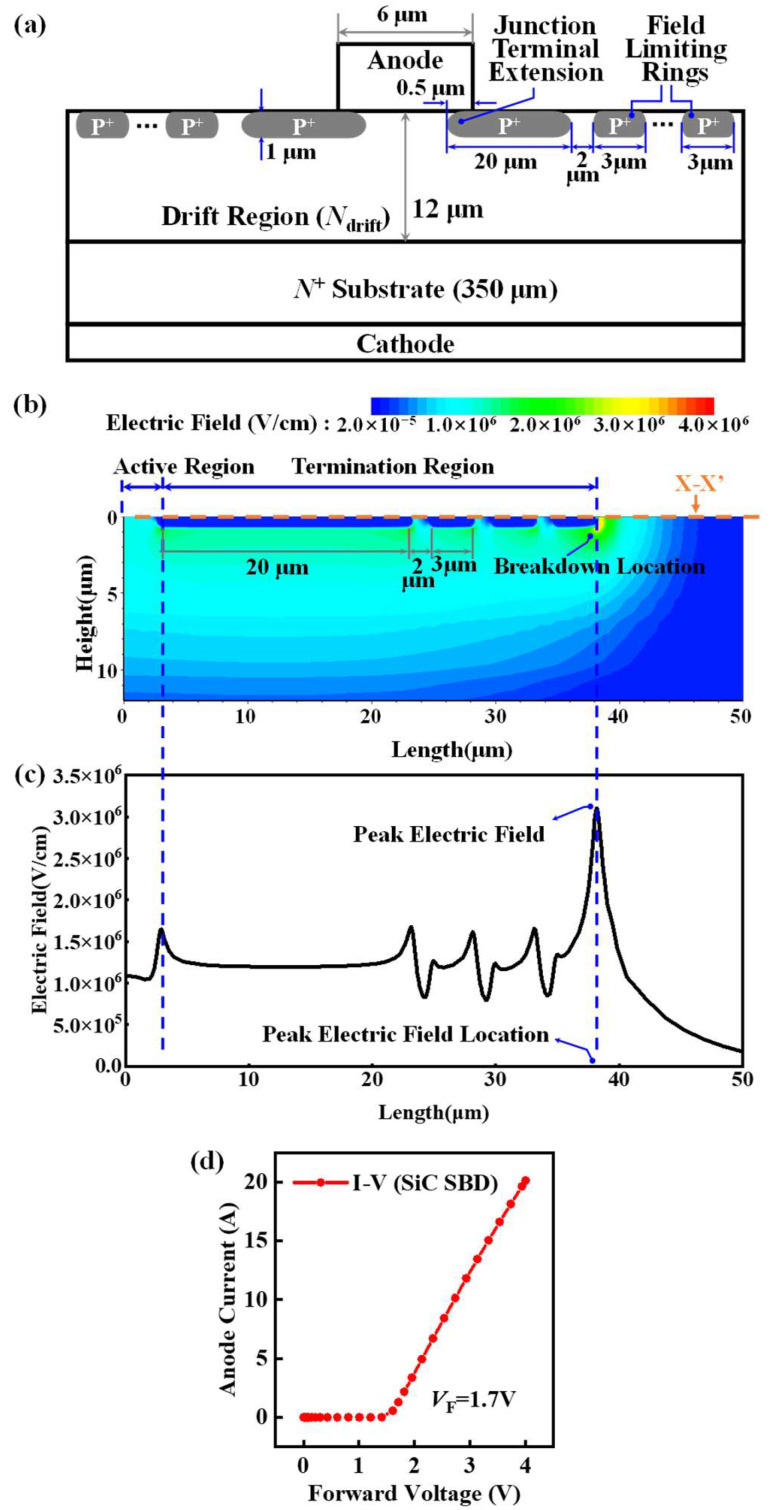
(**a**) Schematic of the SBD studied in this work. JTE and FLR structures were utilized to boost the device’s performance. (**b**) The 2D electric field distribution of the JTE and FLR structures. (**c**) The 1D electric field distribution of the JTE and FLR structures. (**d**) The calibration plot of SiC SBD device characteristics.

This study utilizes TCAD numerical simulation results to construct the dataset. Figure 1 illustrates the schematic of the 4H-SiC SBD used in this research, featuring JTE and FLR, which is widely used in mitigating the electric field concentration effect at the edge of the p-type cell [22]. The substrate exhibits a thickness of 350 μm and a doping concentration of 1 × 10^19^ cm^−3^ [23]. The drift region has a thickness of 12 μm, while FLR width and spacing are 3 μm and 2 μm, respectively [24,25]. Employing a two-dimensional simulation structure significantly reduces the computational workload, enhancing simulation speed. We chose a comprehensive physical model to ensure predictive accuracy while maintaining simulation efficiency. In the simulation process, the basic physical models of 4H-SiC were employed [26,27,28,29,30]. These primarily comprise the Slotboom model for bandgap narrowing, the Auger recombination model, the Shockley–Read–Hall (SRH) model with doping dependence, the avalanche model based on the Okuto equation, the high field saturation model, and the incomplete ionization model. The anisotropic properties of 4H-SiC and barrier-lowering effects were also considered. As shown in Figure 1b,c, breakdown occurs at the endpoint of the limiting ring, effectively mitigating the concentration of the electric field near the PN junction [31]. The line profile of the electric field in Figure 1c is extracted from the X-X′ cutline in Figure 1b, which is the interface between the metal and SiC drift layer. The electric field distribution and voltage peak location are consistent with the results of existing simulation studies [32]. Furthermore, during the simulation process, we calibrated the device characteristics by referencing tape-out results from existing studies. As shown in Figure 1d, the calibrated 1200 V SiC SBD device exhibited a forward conduction voltage (*V*_F_) of 1.7 V, demonstrating high consistency with prior research and, thus, validating the reliability and precision of the dataset [33].

To conduct CNN training, we collected a large dataset. During the simulation, *N*_drift_ varied between 1 × 10^15^ cm^−3^ and 1 × 10^16^ cm^−3^, *N*_A_ varied from 3 × 10^18^ cm^−3^ to 5 × 10^19^ cm^−3^, and the number of field-limiting rings (*n*_FLR_) varied between 1 and 6. *N*_drift_ represents the doping concentration in the drift region; *N*_A_ represents the doping concentration in the p-type region of the terminal structure; and *n*_FLR_ represents the number of field-limiting rings, significantly affecting the breakdown voltage of SiC SBD. The specific values are presented in Table 1.

**Figure 2:**
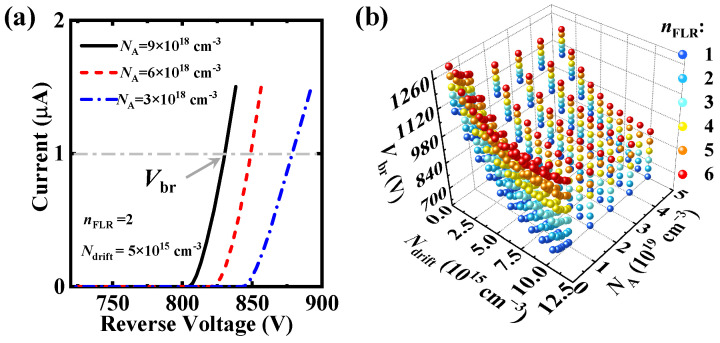
(**a**) Numerically simulated reverse characteristics of the devices with different *N*_A_. *V*_br_ is the reverse voltage at a current of 1 × 10^−6^ A. (**b**) *V*_br_ values of the devices with different *N*_drift_, *N*_A_, and *n*_FLR_.

Figure 2a displays the simulated breakdown characteristics of the SiC SBD under the set parameter group, from which we extracted the *V*_br_ value of the device at the current of 1 μA. In total, 600 sets of *V*_br_ data were collected from numerical simulations, as shown in Figure 2b. The dataset was divided into 60% training, 20% cross-validation, and 20% testing sets. The cross-validation set was not trained and was used to check for overfitting, while the testing set evaluated network performance. As illustrated in Figure 2b, the breakdown voltage of the SiC SBD exhibited an inverse correlation with the *N*_drift_ and *N*_A_. This phenomenon can be attributed to the voltage distribution mechanism under reverse bias conditions, where the applied voltage is shared between the Schottky junction and the PN junctions formed by the field-limiting rings and drift region. The adjustment of *N*_drift_ and *N*_A_ directly modulates the depletion region width, thereby governing the avalanche breakdown characteristics. In contrast, the breakdown voltage demonstrates a positive correlation with the *n*_FLR_. This enhancement mechanism originates from the improved voltage-sharing capability and effective mitigation of electric field crowding at the main junction periphery through implementing multiple field-limiting rings, which optimizes the electric field distribution for device protection.

### 2.2. Analysis of Pre-Training Data

In machine learning optimization processes, it is common to augment the original features with additional, comprehensive ones to provide the model with maximum information. Thus, we conducted data analysis on the initial dataset, experimenting with various random combinations of original features. It was observed that incorporating *n*_FLR_ (X3)/(*N*_drift_ (X1) × *N*_A_ (X2)) as the fourth feature (X4) led to a 2.3% decrease in the average test set error. Achieving such an increase in precision atop an already high level was challenging. This improvement can be attributed to *n*_FLR_ positively correlating with *V*_br_, while the other two parameters exhibited a negative correlation with *V*_br_. The designed composite feature X4 significantly improves the model’s capabilities by integrating the physical relationship between the doping concentration distribution and the terminal structural parameters into the neural network model. Before feeding into the CNN, these features undergo a fully connected layer for data expansion, with the size optimized and adjusted according to the network’s requirements.

### 2.3. Design of Breakdown Voltage Predict Convolutional Neural Network

**Figure 3:**
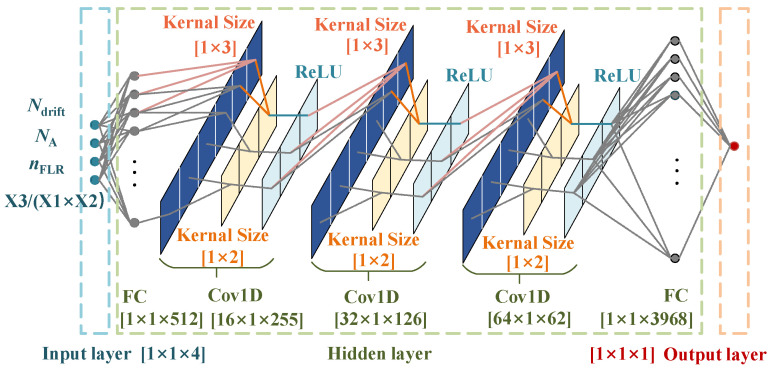
An illustration of the proposed neural network structure design. The network first processes the features with one fully connected layer (FC) with 512 units. The tensor is then fed into three one-dimensional convolutional modules (Cov1D) and connects an FC with 3968 units for data unfolding. Finally, the unfolding data are passed into the output layer, which has an output of size 1 × 1 × 1.

Compared to fully connected neural networks, CNN offers advantages such as local connectivity, weight sharing, and sub-sampling. Figure 3 illustrates the neural network architecture for predicting the SiC SBD *V*_br_, and a three-layer structure suffices to train the format and quantity of the input data used in this study [34]. The kernel is a small matrix used to perform convolution operations on input data to extract local features, and such value significantly influences both the efficiency and the precision of the training process [16]. To achieve the shortest training time and the lowest MSE value, multiple sets of convolutional kernel size and pooling layer kernel size were adopted, with the setup depicted in Figure 3 being selected. Initially, the network processes the features through a single fully connected layer to expand the spatial input dimensions, denoted as H × W (e.g., 1 × 512). Here, H and W are layer hyper-parameters independent of any specific prediction network. Subsequently, the input tensor flows through three convolutional modules, each containing a 1 × 3 filter size convolutional layer.

After normalizing the dataset extracted from TCAD simulations using the Min–Max scaling method, we input the CNN with this normalized dataset to learn the multivariate relationship between *V*_br_ and correlated parameters. Throughout the training process, the network adjusts its weights to minimize discrepancies with TCAD solutions, guided by L2 loss functions. Specifically, the squared difference between the network-generated *V*_br_ predictions (y) and TCAD simulation results ($\hat{y}$) is calculated as follows:

(1) $$L_{\mathrm{CNN}}=\frac{1}{2N}\overset{N}{\underset{1}{\sum }}{\left(y-\hat{y}\right)}^{2}$$

In addition, an optimizer is used in the data training process, which has a mini-batch size of 10 and a momentum value of 0.9. In our study, CNN was compared with several other deep learning methods [35,36] and emerged as the most accurate and efficient model, as shown in the following content.

## 3. Results and Discussion

### 3.1. Prediction Results and Analysis of Breakdown Voltage

**Figure 4:**
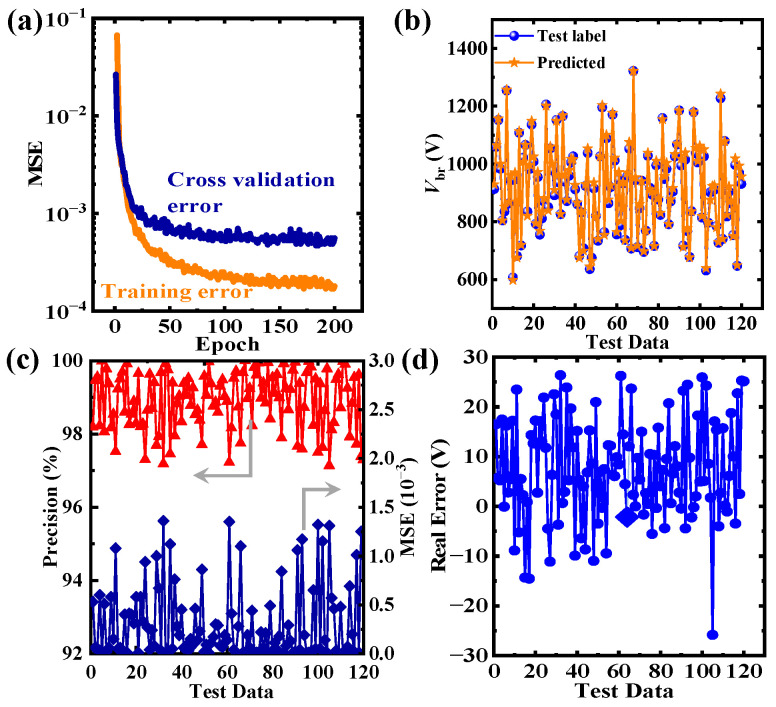
(**a**) The mean MSE of the training set and cross-validation set versus the epoch for CNN training. (**b**) Predicted values and test set label distribution. (**c**) The precision and MSE of the test set and (**d**) real error for the CNN prediction over the 120 test data. The error below 30 V is insignificant compared to the relatively high breakdown voltage (larger than 700 V in this study).

The TCAD simulations required 1.87 × 10^6^ seconds to generate 600 breakdown voltage datasets. However, as shown in Figure 4a, the CNN model achieved a training set MSE of 2 × 10^−4^ following 200 epochs of training completed in 18 s using an AMD Ryzen 3700X (AMD, Santa Clara, CA, USA) processor. This demonstrates that the CNN model can maintain high efficiency in training data incorporating complex physical models. Moreover, the cross-validation set maintains a low gap with the training error in the 200th epoch, indicating non-overfitting calculations. Figure 4b compares predicted *V*_br_ values with test data, revealing negligible discrepancies for each test sample. This similarity suggests that predicted *V*_br_ and actual values (Test label) share nearly identical distributions. Figure 4c displays the prediction precision and MSE of the test set samples, with the CNN achieving over 97% precision and an MSE below 1.5 × 10⁻^3^ across 120 test cases. The low MSE further confirms minimal deviation between the predicted and actual values, with most prediction accuracies reaching 98.5%, underscoring the model’s high efficiency and accuracy. The training process yields a stable model with a strong correlation between *V*_br_ and the corresponding parameters. Figure 4d shows that the actual error between the predicted values and the test set labels remains below 30 V, with the majority of errors confined within 15 V, further demonstrating the model’s capability to reliably predict the breakdown voltage of SiC SBDs.

**Figure 5:**
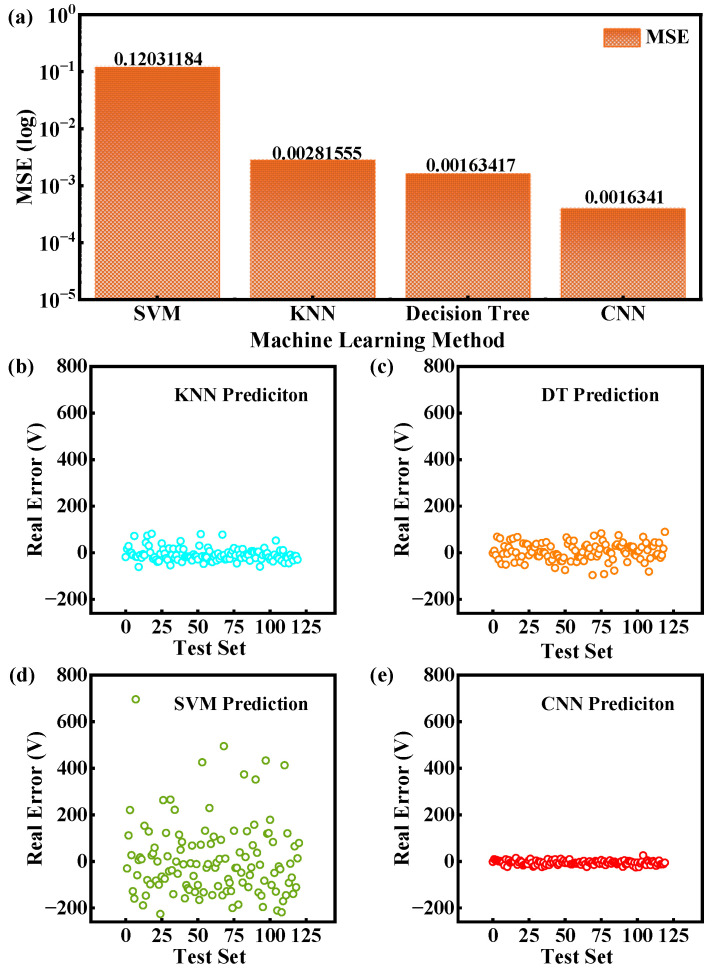
(**a**) The comparison of the MSE of the test set for methods, including SVM, KNN, decision tree, and CNN. (**b**–**e**) Comparative analysis of the prediction errors between KNN, decision tree, SVM, and CNN methods.

Several classical machine learning methods were employed for the comparison with the CNN, including decision tree (DT), k-nearest neighbors (KNNs), and support vector machine (SVM). As shown in Figure 5a, SVM demonstrates the largest MSE in breakdown voltage prediction, indicating significant deviations between predicted and actual values. Although KNN and DT exhibit lower MSE values than SVM, they show noticeable prediction biases. In contrast, the deep learning (DL)-based approach achieves superior accuracy with predictions closely aligned to experimental values, showing the lowest MSE and highest prediction accuracy, which demonstrates excellent data fitting performance.

Figure 5b–d present the error distributions of three machine learning methods and CNNs in predicting breakdown voltages of SiC SBD. The results reveal that while all four methods maintain comparable average errors within 10 V, traditional machine learning methods exhibit significantly greater data dispersion than CNNs. Specifically, SVM shows absolute errors of up to 200 V, with some outliers reaching 600 V. Although KNN and DT show improved error distributions compared to SVM, their errors still fluctuate within 100 V. Remarkably, CNN maintains absolute errors predominantly below 15 V. These findings conclusively demonstrate the superiority of deep learning in SiC SBD prediction, manifested through higher prediction accuracy and substantially reduced error margins.

**Figure 6:**
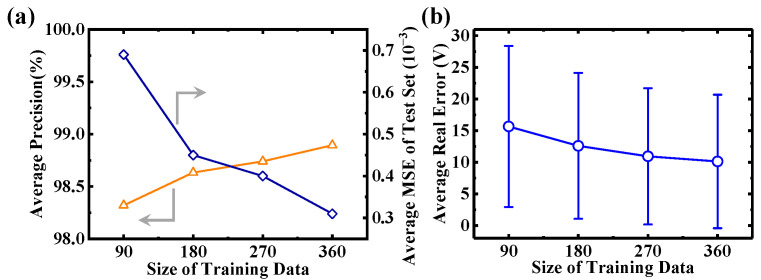
(**a**) The evolution of precision and MSE under the training set sizes of 90, 180, 270, and 360. The reduced MSE and increased precision indicate a more accurate prediction model. (**b**) Average real error versus the size of the training data.

Figure 6 depicts the influence of the training set size on the performance of CNN in this study. As depicted in Figure 6a, average precision increases with larger training set sizes, reaching 98.89% when the training set size is 360. Concurrently, the mean MSE of the test set gradually decreases as the training set size increases, diminishing to 3.10 × 10^−4^ at a size of 360. It is noteworthy that the error bar of the test set also decreases with the increasing training set size, albeit at a slower rate, stabilizing at around 10.13 at a size of 360. While a larger training set improves model accuracy and reduces MSE, the diminishing returns suggest caution in scaling up without discretion. This is relevant for applications like SiC SBD devices, where performance predictions correlate broadly with specific parameters to avoid unnecessary computational resource expenditure.

### 3.2. Prediction Results and Analysis of I-V Forward Conduction Curves

Our model based on deep learning allowed us to predict not only the breakdown voltage value but also the forward *I-V* curve of SiC SBDs. For curve prediction, each curve was divided into 250 points with 600 sets of data as the total input. In addition, the network architecture used for predicting both the *I-V* curve and the breakdown voltage was identical. The difference is that the final layer of the network is a one-dimensional FC with a size of 1 × 1 × 250 for *I-V* curve prediction. This indirectly demonstrates the transferability and applicability of neural networks.

**Figure 7:**
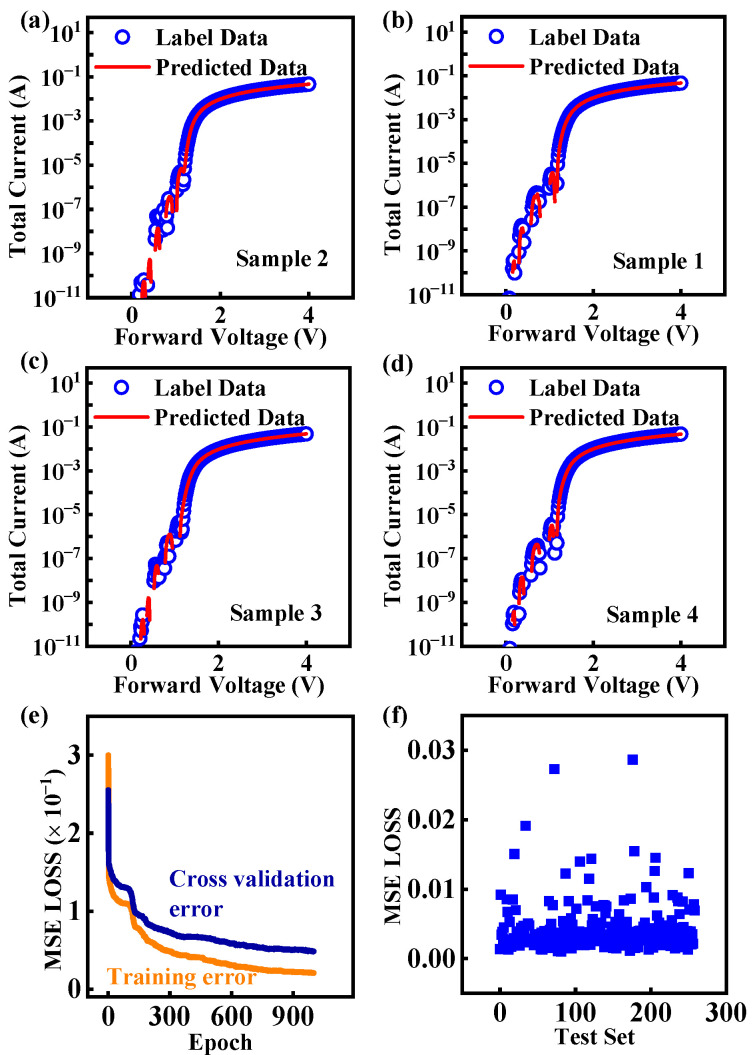
The predicted *I-V* curves of SiC SBD devices in this work. (**a**–**d**) The comparison graphs of the original curves and predicted curves of four groups of data randomly selected from the test set. (**e**) The MSE value of the training set and the cross-validation set of each iteration in the network training process. (**f**) The MSE loss function values of the test set.

Figure 7a–d present four randomly selected samples from the prediction results, showing that the predicted values are almost identical to the original data. Figure 7e illustrates the declining trend of training loss and cross-validation loss during the training process. The results show that after about 1000 iterations of the results, the model achieves an MSE loss of less than 6 × 10^−2^ on both the training set and the cross-validation set. Figure 7f shows that the MSE loss on the test set is primarily concentrated at around 1 × 10^−3^, exhibiting high precision for deep learning-based models in predicting the forward *I-V* curves of SiC SBDs.

## 4. Conclusions

In summary, a deep learning approach based on CNN was implemented to predict the *V*_br_ and forward *I-V* curves of SiC SBDs. The developed model achieved an average prediction accuracy of 99%, with the majority of predicted breakdown voltage values exhibiting deviations within 15 V from the reference values. Comparative error analysis demonstrated that the proposed modeling approach based on CNN outperformed conventional machine learning techniques in terms of both accuracy and stability. Furthermore, the CNN-predicted *I-V* curves demonstrated excellent agreement with target profiles, achieving an MSE below 1 × 10⁻^3^. Importantly, the prediction model also supports a linear variation in device structural parameters and is applicable to SiC SBD devices of any voltage rating, which significantly enhance the efficiency of industrial and academic researchers in designing SiC SBD devices. This work validates the feasibility of artificial intelligence and experimental data for modeling destructive experiments on power devices, effectively circumventing the simulation inefficiency caused by excessively low leakage currents and eliminating the need for extensive destructive testing during breakdown voltage characterization.

## Figures and Tables

**Table 1:** Values of *N*_drift_, *N*_A_, and *n*_FLR_ used in the simulation.

Parameters	Values
*N*_drift_ (cm^−3^)	1 × 10^15^, 2 × 10^15^, 3 × 10^15^, 4 × 10^15^, 5 × 10^15^, 6 × 10^15^, 7 × 10^15^, 8 × 10^15^, 9 × 10^15^, and 1 × 10^16^
*N*_A_ (cm^−3^)	3 × 10^18^, 5 × 10^18^, 6 × 10^18^, 7 × 10^18^, 9 × 10^18^, 1 × 10^19^, 2 × 10^19^, 3 × 10^19^, 4 × 10^19^, and 5 × 10^19^
*n*_FLR_	1, 2, 3, 4, 5, and 6

## Data Availability

The original contributions presented in this study are included in the article. Further inquiries can be directed to the corresponding author.

## Abbreviations

The following abbreviations are used in this manuscript:

CNNs	Convolutional Neural Networks
Cov1D	One-Dimension Convolutional Modules
DT	Decision Tree
FC	Fully Connected Layer
FLR	Field-Limiting Ring
JTE	Junction Termination Extension
KNN	K-Nearest Neighbor
SVM	Support Vector Machine
